# Progression to Pseudomonas Osteomyelitis Following Pin Tract Infection After Percutaneous Fixation of a Pediatric Supracondylar Humerus Fracture

**DOI:** 10.7759/cureus.87058

**Published:** 2025-06-30

**Authors:** Ayami Kitagawa, Dai Otsuki, Chikahisa Higuchi, Daisuke Tamura

**Affiliations:** 1 Department of Rehabilitation Medicine, Osaka Women’s and Children’s Hospital, Izumi, JPN; 2 Department of Orthopedic Surgery, Osaka Women’s and Children’s Hospital, Izumi, JPN

**Keywords:** osteomyelitis, pediatric orthopedic surgery, pin site infections, postoperative complication, pseudomonas aeruginosa, supracondylar humerus fracture

## Abstract

Supracondylar humerus fractures are common in children, and percutaneous pinning is the standard treatment. Infection rates are low, and *Pseudomonas aeruginosa* osteomyelitis is rarely reported. A 4-year-old boy underwent percutaneous pinning for a supracondylar humerus fracture. Five weeks postoperatively, redness appeared at the medial pin site and that wire was removed; at six weeks the lateral site later developed granulation tissue, but no antibiotics were prescribed. At eight weeks he developed a 39°C fever. MRI on postoperative day 60 suggested osteomyelitis, and CT on day 65 demonstrated a cortical defect in the posterolateral distal humerus. Surgical debridement confirmed *P. aeruginosa* infection. Culture-directed therapy with intravenous cefepime plus ciprofloxacin - initiated 30 days after the first local signs and continued for five weeks - was followed by six months of oral ciprofloxacin; elbow function recovered to 0°-150° despite a residual cortical defect. This case shows that delayed antibiotics can allow a superficial pin-site infection to progress to deep osteomyelitis, and underscores that early recognition of pin-site changes with prompt antimicrobial therapy is essential to prevent deep infection and preserve function.

## Introduction

Supracondylar humerus fractures are one of the most common fractures in children, and percutaneous pinning is widely used as the standard method of fixation [1] . Standard peri-operative care typically includes sterile preparation, prophylactic antibiotics, and wire removal at about four weeks [2]. Reported complications-ulnar nerve injury, pin migration, malunion, and infections are overall uncommon [3]. Postoperative pin-site infections remain relatively low, reported at around 1 %, and progression to deep infection such as osteomyelitis or septic arthritis is exceedingly rare, occurring in approximately 0.2 % of cases [4]. *Pseudomonas aeruginosa* is an especially unusual pathogen: since the pediatric case reported by Wegner et al. in 2017, no additional instances have been published [5]. Here, we report a case of osteomyelitis that developed from a pin-site infection during postoperative management following percutaneous pinning for a supracondylar humerus fracture in a 4-year-old boy; delayed initiation of antibiotics allowed the superficial infection to progress, yet timely debridement and culture-directed therapy enabled recovery of elbow motion to a level that no longer limited activities of daily living (ADL). This case therefore adds to the scant literature and underscores the need for vigilance when late pin-site changes appear.

## Case presentation

**Figure 1:**
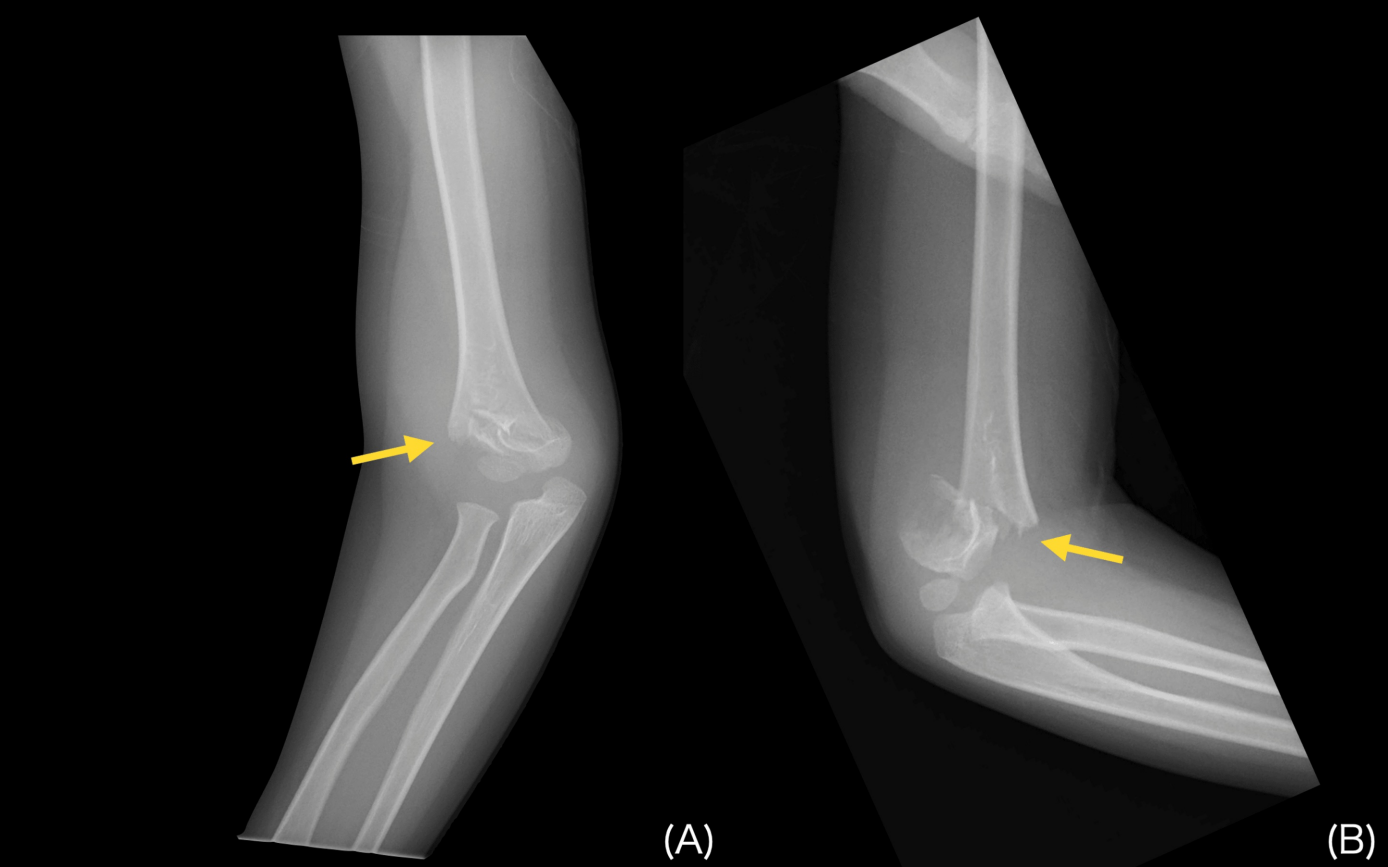
Initial injury radiographs of a displaced supracondylar humerus fracture Initial plain radiographs of the right elbow revealing a displaced supracondylar humerus fracture: anteroposterior view (A) and lateral view (B). Yellow arrows indicate the fracture lines.

A 4-year-old boy fell out of a car while still in his child seat and sustained trauma to his right elbow. He was taken to a local orthopedic clinic (Hospital A), where plain radiographs (Figure 1) revealed a supracondylar fracture of the right humerus.

**Figure 2:**
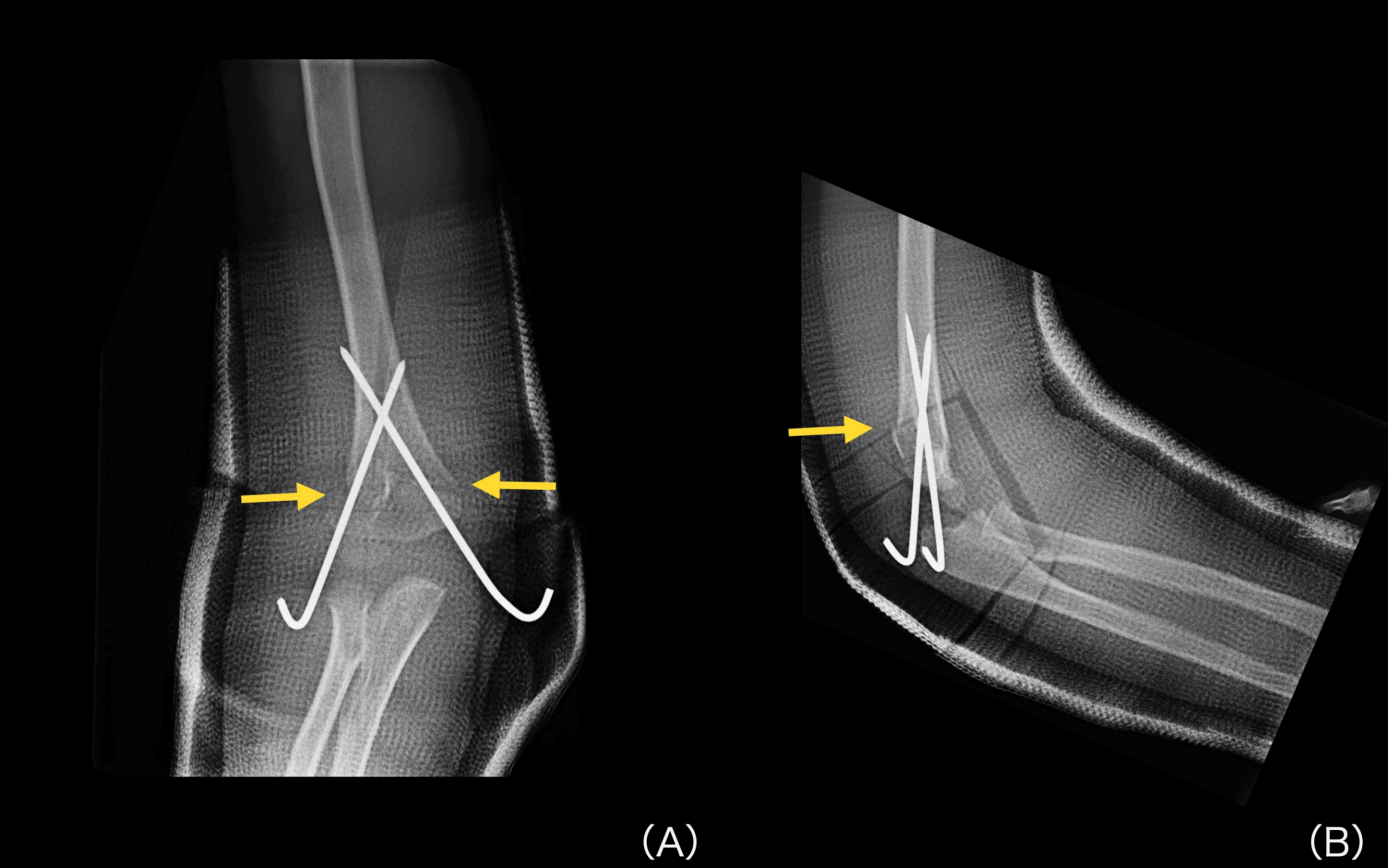
Immediate postoperative radiographs after percutaneous pinning of a supracondylar humerus fracture Immediate postoperative plain radiographs of the right elbow after percutaneous pinning, showing crossed Kirschner-wire fixation of the supracondylar humerus fracture: anteroposterior view (A) and lateral view (B). Yellow arrows indicate the fracture lines.

He underwent percutaneous pinning for fracture fixation (Figure 2).

Details regarding the interval between injury and surgery, the condition of the skin, and any peri-operative antibiotic prophylaxis were not documented in the records provided by the referring hospital. Postoperative follow-up was performed at Hospital B. At five weeks postoperatively, redness was observed around the medial pin site, and the medial wire was removed. At six weeks, when the cast was removed, granulation tissue was noted at the lateral pin site, raising suspicion of infection; the lateral wire was also removed at that time. However, no antibiotics were administered. The patient was later referred to Hospital C for rehabilitation purposes. However, due to worsening granulation tissue at the lateral pin site, he was referred again to the plastic surgery department at Hospital B. On postoperative day 60, he developed a fever of 39°C. Pin-site swab cultures were obtained immediately before initiating intravenous ceftriaxone (CTRX). Thereafter, CTRX was administered in Hospital B’s orthopedic department. Blood tests showed a white blood cell count of 5,700/μL and a C-reactive protein (CRP) level of 4.84 mg/dL (Table 1). 

**Table 1:** Laboratory findings at onset of 39°C fever Blood tests obtained when the patient developed a 39°C fever and intravenous antibiotics were initiated; no marked elevation of inflammatory markers was observed. WBC: white blood cell; CRP: C-reactive protein

Parameter	Patient Value	Reference Value
WBC (×10³/µL)	5.7	5.0–8.5
CRP (mg/dL)	4.84	< 0.3

**Figure 3:**
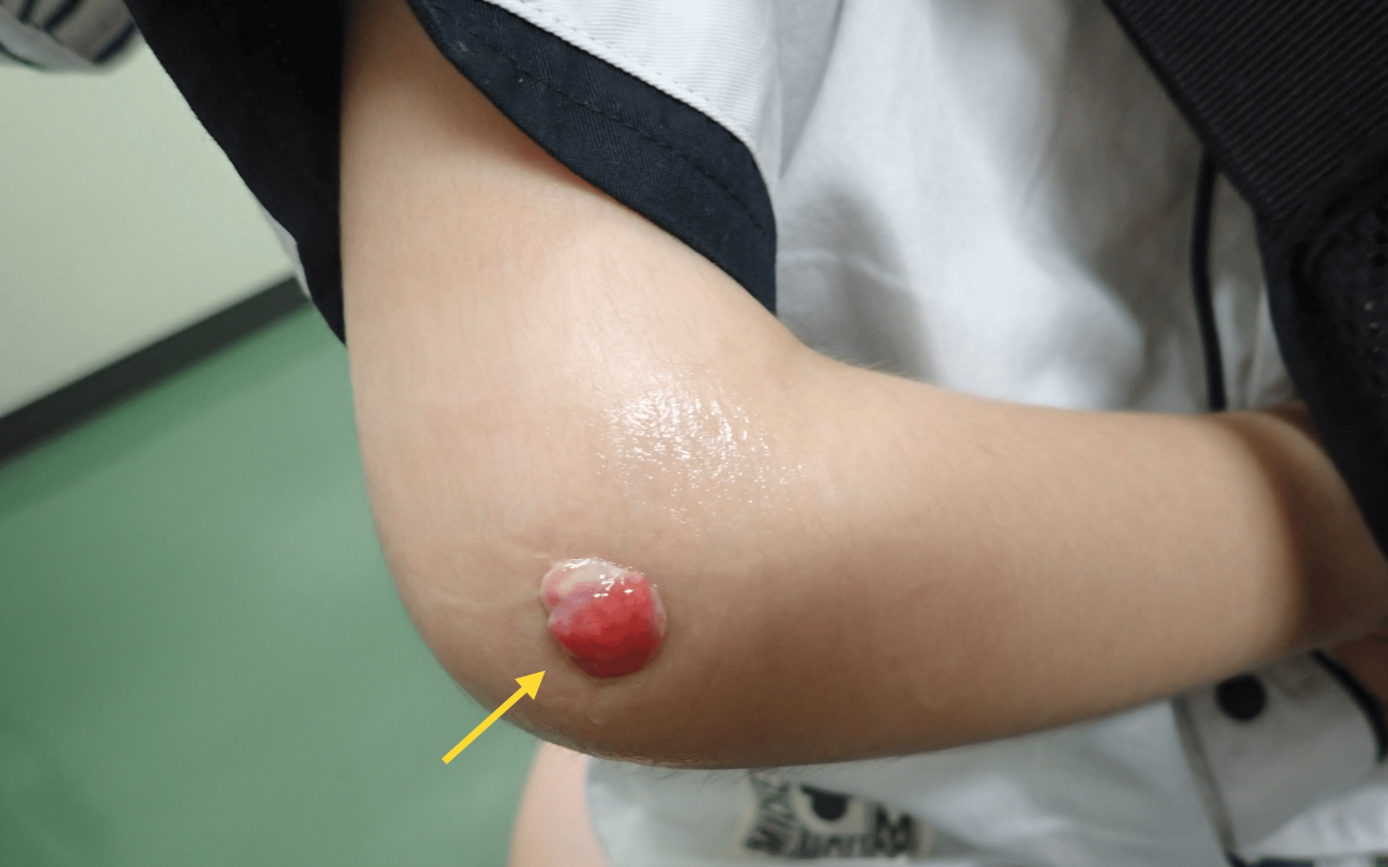
Clinical photograph showing granulation tissue at the lateral pin site Granulation tissue forming at the lateral wire removal site of the right elbow (yellow arrow). WBC: white blood cell; CRP: C-reactive protein

Culture of the pin site was negative. However, magnetic resonance imaging (MRI) performed on postoperative day 60 revealed findings suggestive of pyogenic osteomyelitis, and the patient was referred to our hospital, where he presented on postoperative day 65. At the initial visit, granulation tissue and exudate were noted at the lateral pin site of the elbow joint (Figure 3). 

Blood tests showed a white blood cell count of 6,000/μL and a mildly elevated CRP level of 1.45 mg/dL (Table 2).

**Table 2:** Laboratory findings at initial presentation to our hospital Blood tests obtained on referral to our hospital, showing a mild elevation of inflammatory markers. WBC: white blood cell; CRP: C-reactive protein

Parameter	Patient Value	Reference Value
WBC (×10³/µL)	6.0	5.0–8.5
CRP (mg/dL)	1.45	< 0.3

**Figure 4:**
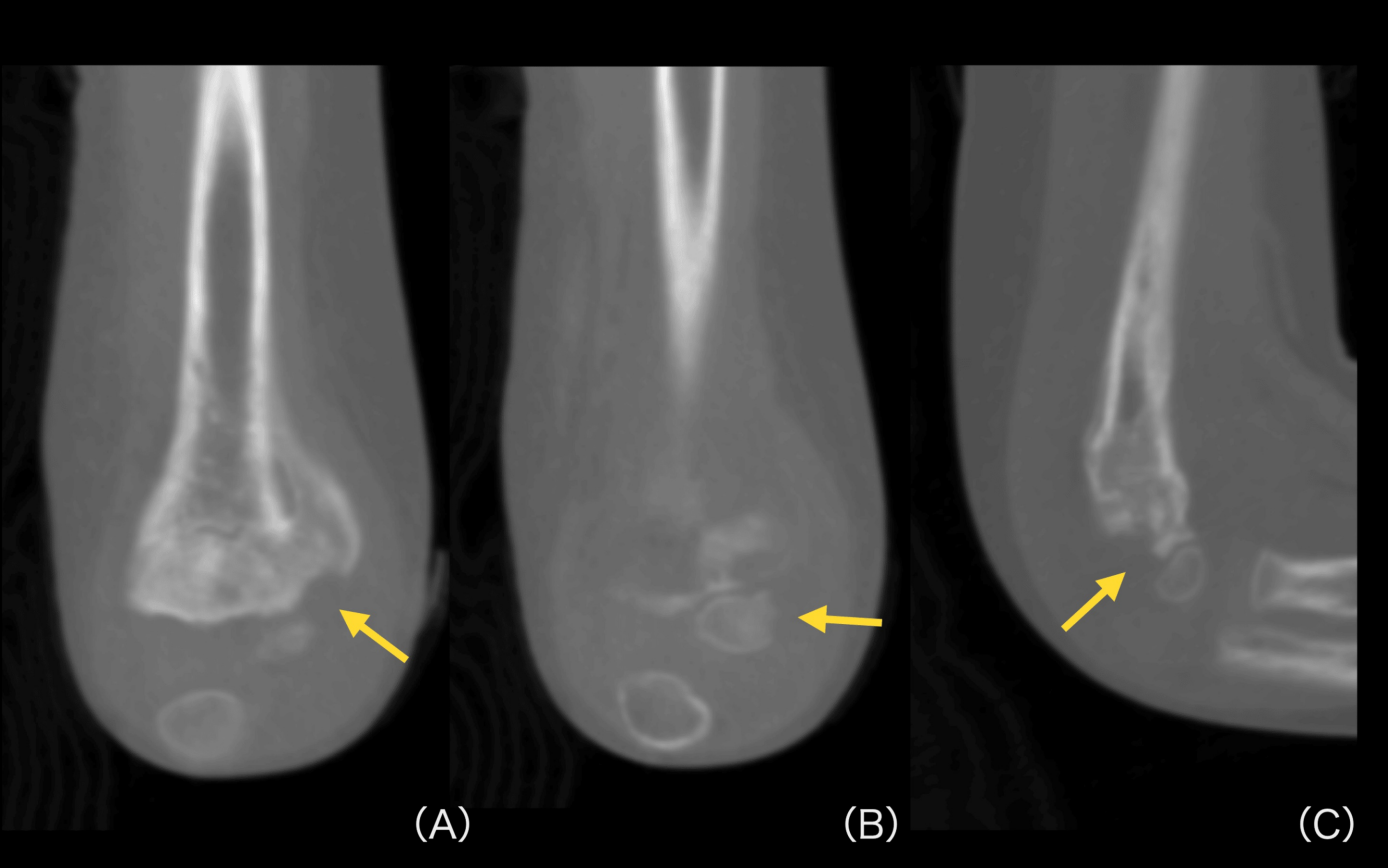
Coronal and sagittal CT demonstrating cortical breach and bone defect Coronal and sagittal CT images of the right elbow demonstrating a cortical bone defect in the posterolateral distal humerus: coronal view at the level of the defect (A), coronal view at an adjacent level (B), and sagittal view (C). Yellow arrows indicate the bone defect; the loss of cortical continuity confirms infective bone destruction rather than malreduction of the fracture or unossified cartilage. These findings correspond to the cavitary lesion visualized on three-dimensional reconstruction (Figure 5).

**Figure 5:**
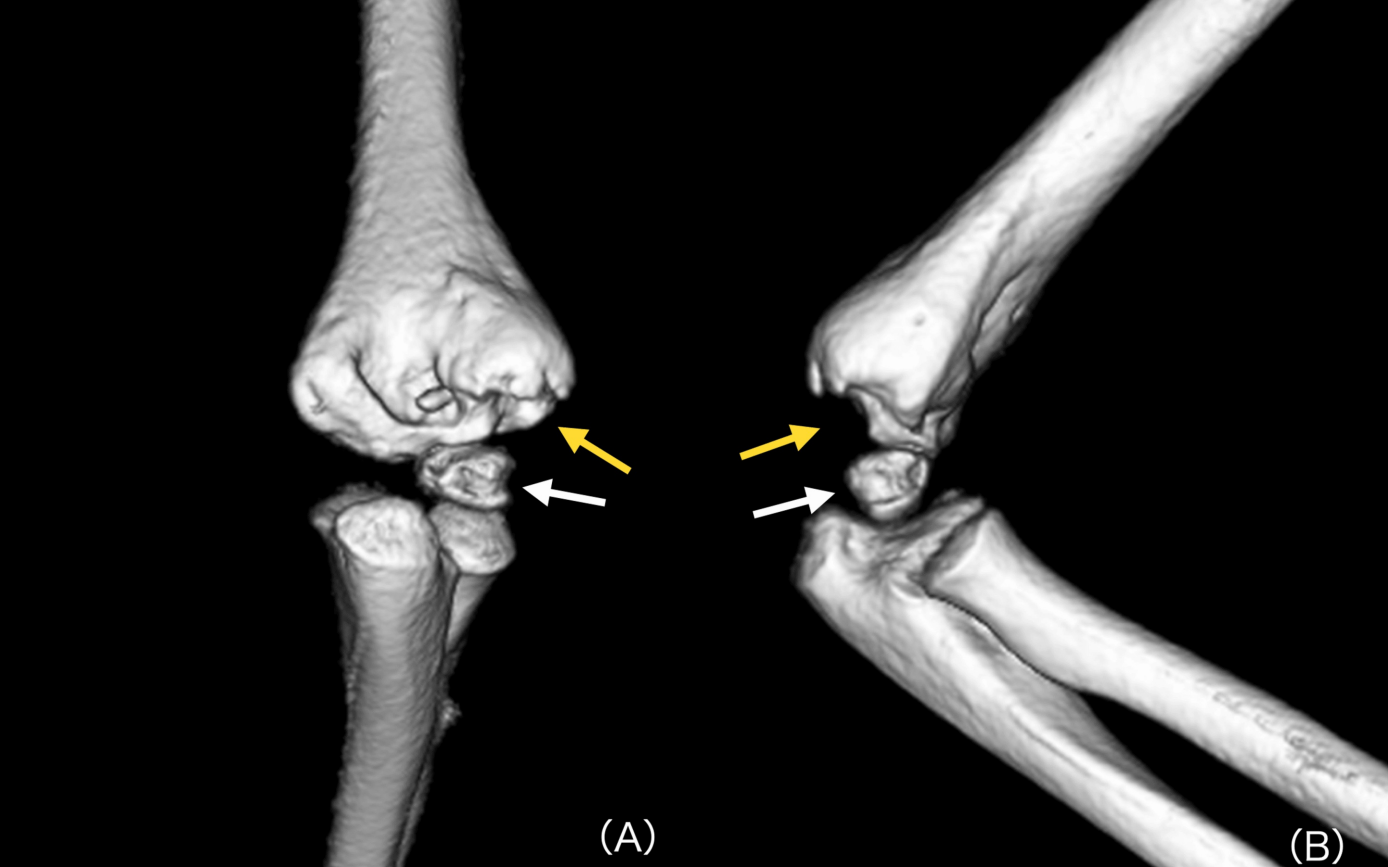
Three-dimensional CT highlighting cortical bone defects Three-dimensional CT reconstruction of the right elbow demonstrating cortical bone defects in the posterolateral distal humerus and capitellum: anteroposterior view (A) and lateral view (B). Yellow arrows indicate the defect in the distal humerus, and white arrows indicate the defect in the capitellum.

**Figure 6:**
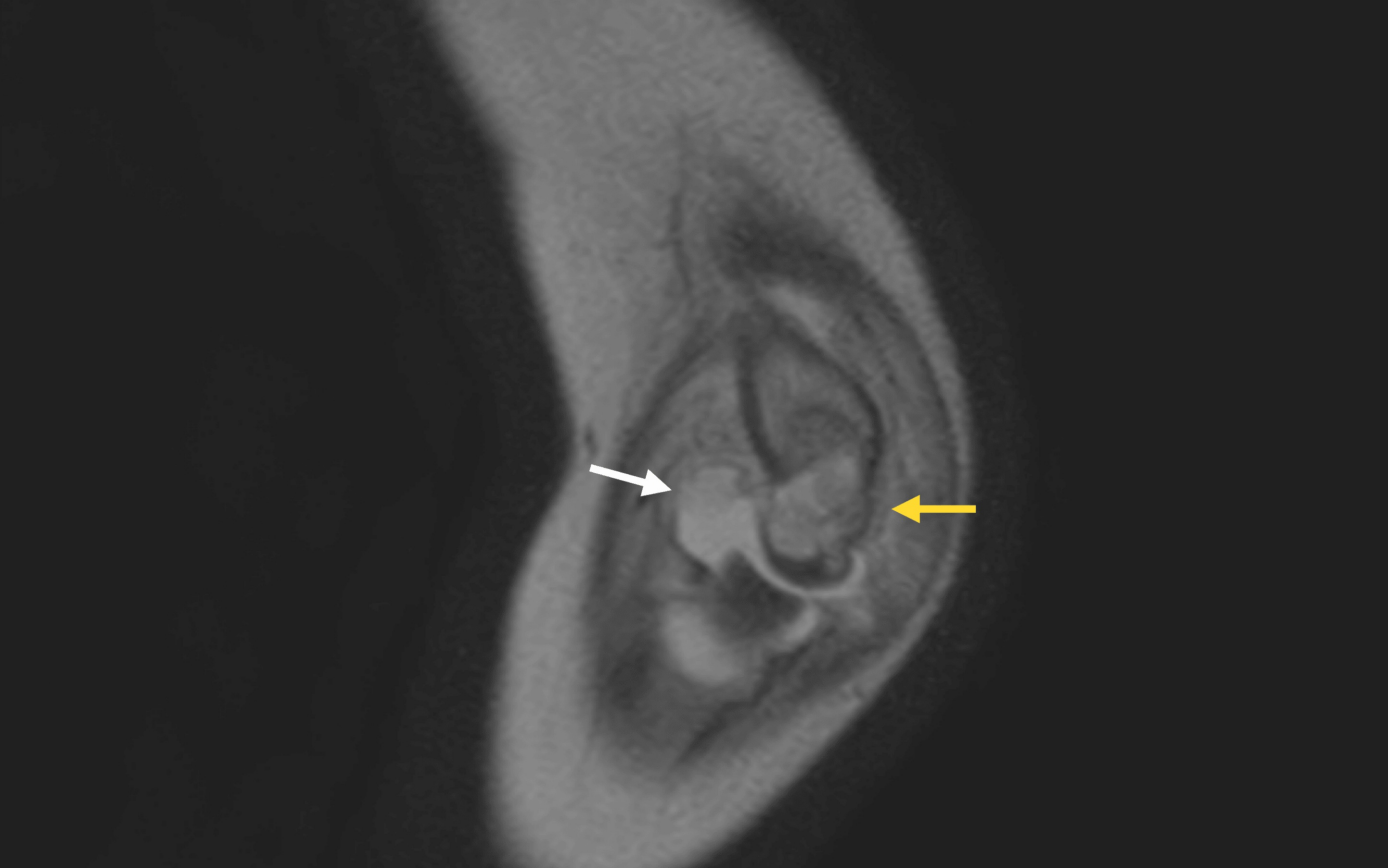
Coronal MRI indicating osteomyelitis of the distal humerus Coronal MRI of the right elbow showing joint effusion (white arrow) and marrow hyperintensity within the distal humerus (yellow arrow), findings consistent with osteomyelitis.

Although the white-blood-cell count remained within the normal range, the elevated CRP level suggested an ongoing deep-seated infection. Plain radiographs and computed tomography (CT) obtained on postoperative day 65 revealed a bone defect in the posterolateral aspect of the humerus (Figures 4-5), and MRI showed joint effusion and a high-signal-intensity area within the bone marrow (Figure 6). 

**Figure 7:**
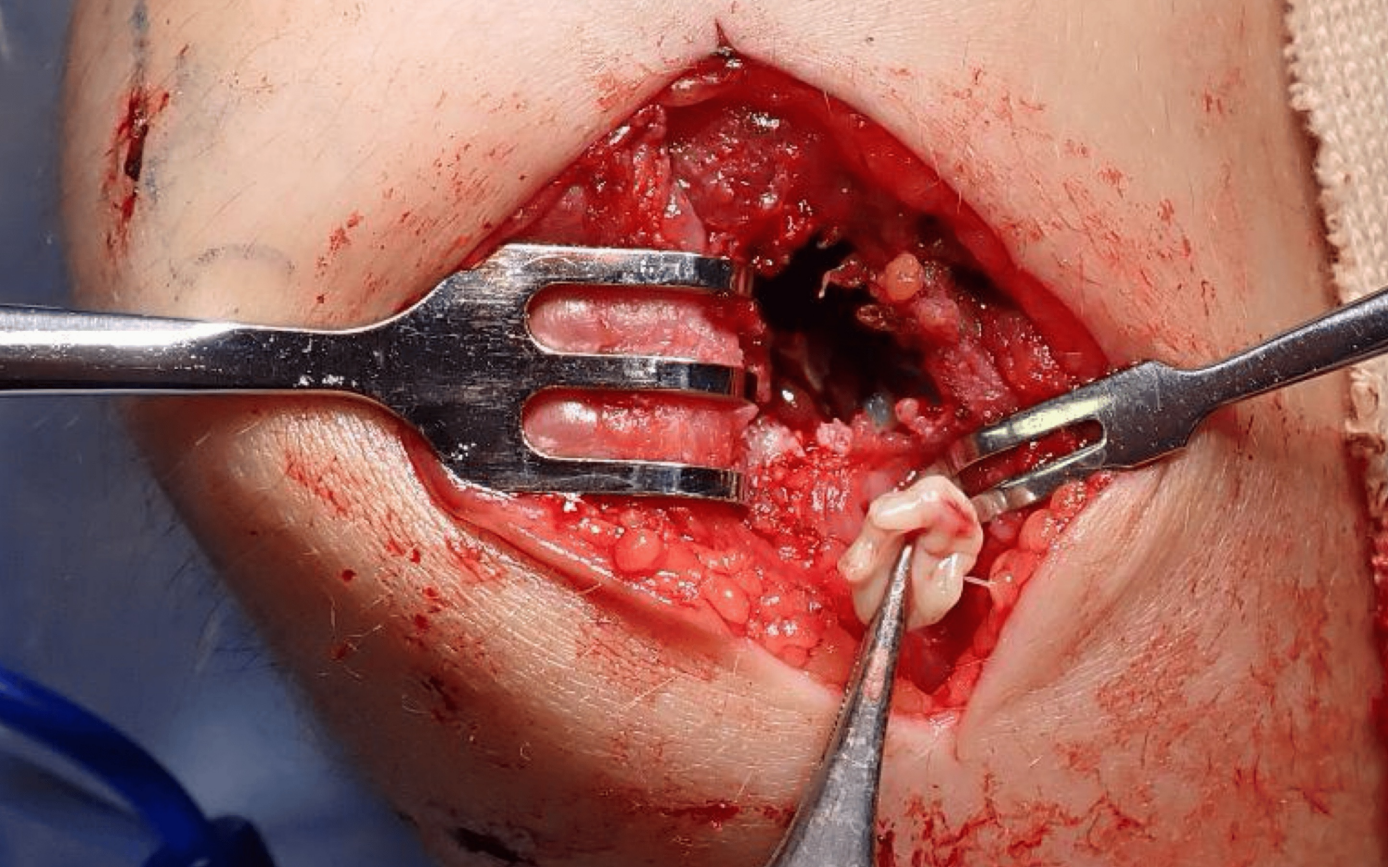
Intra-operative view of posterolateral humeral cavity Intra-operative photograph of the right elbow showing a cavity in the posterolateral distal humerus surrounded by granulation tissue.

**Figure 8:**
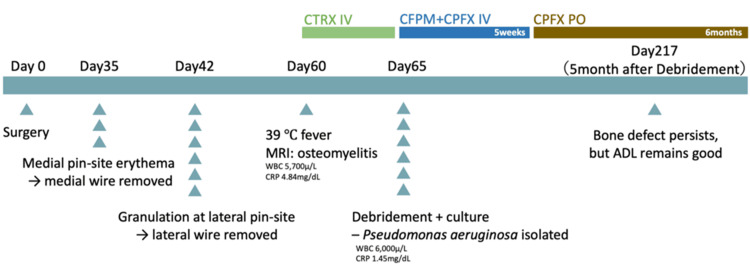
Chronological timeline of clinical events, laboratory data, imaging, and treatment The timeline summarizes key milestones from initial pinning (Day 0) to the 5-month postoperative follow-up. Superficial pin-site changes appeared at Day 35 (medial erythema) and Day 42 (lateral granulation); wires were removed accordingly. A 39°C fever on Day 60, with laboratory values (WBC 5,700 µL, CRP 4.84 mg/dL) and MRI suggestive of osteomyelitis, prompted hospital referral. Debridement and culture on Day 65 isolated *Pseudomonas* ​​​​​​*aeruginosa* (WBC 6,000 µL, CRP 1.45 mg/dL). Intravenous CTRX was followed by 5 weeks of CFPM + CPFX IV and 6 months of oral CPFX. At 5 months post-debridement (Day 217) a cortical bone defect persisted, but activities of daily living were unrestricted. CTRX: ceftriaxone; CFPM: cefepime; CPFX: ciprofloxacin

Based on these findings, a diagnosis of pyogenic osteomyelitis was made, and surgical debridement was performed. Intraoperatively, the articular cartilage of the capitellum was barely preserved. In contrast, the posterolateral humerus exhibited a significant cavity consistent with a bone defect. The granulation tissue surrounding the cavity was debrided (Figure 7), and culture of the specimen revealed *P. aeruginosa*. In light of the susceptibility profile, the patient received a five-week course of intravenous cefepime (CFPM) plus ciprofloxacin (CPFX), followed by six months of oral CPFX. Intraoperatively, elbow extension was found to place mechanical stress on the radiocapitellar cartilage, so rehabilitation included restriction of elbow extension for one month postoperatively. At the final follow-up, five months after the debridement surgery, plain radiographs showed that the bone defect remained. However, active elbow range of motion had recovered to 0° of extension and 150° of flexion, a level that no longer interfered with ADL. A chronological summary of the clinical course is presented in Figure 8.

## Discussion

We reviewed factors related to infection following supracondylar humerus fractures, including preoperative preparation, postoperative management, and the duration of wire retention.

First, there is no consensus on the optimal preoperative skin preparation method for supracondylar humerus fractures. Studies have shown no significant difference in infection rates between the use of povidone-iodine and alcohol-based solutions [3]. Iobst et al. also reported that performing surgery without sterile gowns and using only sterile gloves, or preparing the surgical field with sterile towels, did not result in increased infection rates [6]. Furthermore, some studies suggest that the use or omission of preoperative antibiotics before surgery does not significantly affect infection rates [3].

Regarding postoperative management, Lu et al. compared infection rates based on the frequency of pin site care-daily, every other day, or once a week-and found no significant differences [7]. They also stated that clinical findings at the pin site, such as pain, redness, or discharge, may influence the timing and decision to initiate antibiotic therapy. In particular, they regarded early initiation of antibiotics in the presence of pain at the pin site as important [7].

In clinical practice, prolonged retention of wires is often associated with an increased risk of infection, and wires are commonly removed around 28 days postoperatively. However, this timing is largely based on empirical experience, and there is no strong supporting evidence [2]. In fact, some reports describe infections occurring even when wires were removed within 28 days, while others have shown uneventful outcomes despite removal after 28 days [8]. Jones et al. reported that removing the cast within four weeks postoperatively does not increase the risk of infection or refracture [2]. Based on this, early cast removal-before 28 days-is considered beneficial. Prolonged immobilization with casting is generally not recommended, except in severe cases such as comminuted fractures.

In this case, signs of infection at the pin sites had already been observed by 5 to 6 weeks postoperatively, when the wires were removed. If antibiotics had been initiated at that point, progression to osteomyelitis might have been prevented. While regular pin site care and monitoring for signs of infection are important, frequent checks can be challenging in pediatric patients. In clinical settings, extended immobilization for more than four weeks may be necessary in cases such as comminuted fractures. In such situations, creating a small window over the pin site in the cast around postoperative week 4 may be a useful strategy to allow continued immobilization while enabling pin site care and observation. MRI performed on postoperative day 60 showed diffuse marrow T2 hyperintensity extending well beyond the fracture line and joint effusion; together with fever, pin-site granulation and progressive pain, these findings favored a diagnosis of acute osteomyelitis rather than oedema associated with normal fracture healing. The initial pin-site swab obtained at the referring hospital yielded no growth, a result that is not uncommon when sampling superficial granulation tissue after wire removal. Such a negative culture does not exclude a deeper, smoldering infection; indeed, curettage at our institution subsequently isolated *P. aeruginosa* from the granulation tissue surrounding the cavity, confirming that the superficial focus had resolved while a deep focus persisted.

Among the pathogens associated with infections following percutaneous pinning, methicillin-sensitive *Staphylococcus aureus* (MSSA) is the most common, followed by *P. aeruginosa*, *Streptococcus*, and *Staphylococcus epidermidis* [9]. Reports of *Pseudomonas aeruginosa* osteomyelitis after percutaneous pinning remain exceedingly scarce. A comprehensive PubMed search (2020 - 2025) using the terms “*Pseudomonas*”, “supracondylar humerus”, “pin-site infection”, and “Kirschner wire” yielded no new pediatric cases, indicating that the single case reported by Wegner et al. in 2017 is still the most recently published example [5]. The present report therefore represents one of the very few documented instances. *P. aeruginosa* thrives in moist environments such as around water sources [10]. And it is considered more likely to be involved in cases where patients miss follow-up appointments or fail to properly manage cast care at home-such as allowing the cast to remain wet or not addressing sweat accumulation [5]. In the present case, prolonged cast immobilization and the fact that the treatment period occurred during the summer may have created a moist environment conducive to *P. aeruginosa* growth. From an infection prevention perspective, it is important to educate patients and families about proper cast care, including protecting the cast during showers and avoiding excessive sweating.

Limitations of this report include its single-case design, the incomplete peri-operative data provided by the referring hospital, the lack of information on caregiver education and cast-care-follow-up at the outside facility, and the unknown dosage of the prophylactic antibiotic reportedly administered there; consequently, any generalization of these findings should be made with caution.

## Conclusions

We experienced a rare case of osteomyelitis caused by *P. aeruginosa* following pin site infection after percutaneous pinning of a pediatric supracondylar humerus fracture. While it is difficult to completely prevent postoperative infections, reducing the risk may be possible by aiming for wire removal around 4 weeks postoperatively and regularly assessing pin site conditions. In particular, when signs of infection such as pain, redness, or discharge are observed, early wire removal and prompt antibiotic treatment are important to prevent progression to severe infections such as osteomyelitis. Despite a residual cortical defect, timely debridement and culture-directed antibiotics led to full elbow motion and unrestricted ADL.
